# Investigating conversational dynamics in triads: Effects of noise, hearing impairment, and hearing aids

**DOI:** 10.3389/fpsyg.2024.1289637

**Published:** 2024-04-12

**Authors:** Eline Borch Petersen

**Affiliations:** WS Audiology, Lynge, Denmark

**Keywords:** hearing loss, communication, hearing aids, noise, conversational dynamics

## Abstract

Communication is an important part of everyday life and requires a rapid and coordinated interplay between interlocutors to ensure a successful conversation. Here, we investigate whether increased communication difficulty caused by additional background noise, hearing impairment, and not providing adequate hearing-aid (HA) processing affected the dynamics of a group conversation between one hearing-impaired (HI) and two normal-hearing (NH) interlocutors. Free conversations were recorded from 25 triads communicating at low (50 dBC SPL) or high (75 dBC SPL) levels of canteen noise. In conversations at low noise levels, the HI interlocutor was either unaided or aided. In conversations at high noise levels, the HI interlocutor either experienced omnidirectional or directional sound processing. Results showed that HI interlocutors generally spoke more and initiated their turn faster, but with more variability, than the NH interlocutors. Increasing the noise level resulted in generally higher speech levels, but more so for the NH than for the HI interlocutors. Higher background noise also affected the HI interlocutors’ ability to speak in longer turns. When the HI interlocutors were unaided at low noise levels, both HI and NH interlocutors spoke louder, while receiving directional sound processing at high levels of noise only reduced the speech level of the HI interlocutor. In conclusion, noise, hearing impairment, and hearing-aid processing mainly affected speech levels, while the remaining measures of conversational dynamics (FTO median, FTO IQR, turn duration, and speaking time) were unaffected. Hence, although experiencing large changes in communication difficulty, the conversational dynamics of the free triadic conversations remain relatively stable.

## 1 Introduction

Living with hearing loss affects not only the ability to hear but also the way a person interacts with others in communication situations. Communication is a core activity in our everyday lives and relies on the ability to switch rapidly and continuously between listening and talking. Conversations are complex interactions consisting of linguistic, auditory, and visual components that can be adapted to overcome communication challenges. For example, it has been known for more than 100 years that humans adapt their speech when communicating in noise (Lombard speech) by increasing the intensity, pitch, and duration of words (Lombard, 1911; Junqua, 1996). Similarly, it has been observed that when communicating with an elder hearing impaired (HI) interlocutor, younger normal-hearing (NH) interlocutors speak louder in quiet and noisy situations, reduce their articulation rate, and alter the spectral content of their speech (Hazan and Tuomaine, 2019; Sørensen et al., 2019; Beechey et al., 2020b; Petersen et al., 2022). These changes suggest that the NH interlocutors adapt their speech to alleviate the communication difficulty experienced by their HI communication partner. Furthermore, it has been observed that when providing the HI interlocutors with hearing aids (HAs), the HI interlocutors reduce the duration of their utterances (inter-pausal units), speak faster (higher articulation rate), and decrease their speech level (Beechey et al., 2020a; Petersen et al., 2022). Additionally, when the HI interlocutor is aided, the NH interlocutor also decreases their speech level despite not directly experiencing any alteration in the communication difficulty (Beechey et al., 2020a; Petersen et al., 2022).

Another aspect of a conversation is the interactive turn-taking between interlocutors and the timing of the turn-starts, denoted as floor-transfer offsets (FTOs). Despite taking at least 600 ms to physically produce a verbal response (Indefrey and Levelt, 2004; Magyari et al., 2014), turns are generally initiated after a short pause of around 200 ms (Stivers et al., 2009), indicating that turn-ends must be predicted to initiate a fast response (Bögels et al., 2015; Gisladottir et al., 2015; Levinson and Torreira, 2015; Barthel et al., 2016; Corps et al., 2018). Impaired hearing causes the talker to initiate their turns in a less well-timed manner, evident from a larger variability in their FTOs compared to NH interlocutors (Sørensen, 2021; Petersen et al., 2022). When receiving HA amplification, the FTOs of HI interlocutors become less variable, indicating that some of their communication difficulty is relieved. This allows them to provide more well-timed verbal responses (Petersen et al., 2022).

From the studies referred to above, results showed that the added communication difficulty experienced by HI interlocutors affected not only the dynamics of their own speech but also of their NH conversational partner (Hazan and Tuomaine, 2019; Beechey et al., 2020b; Sørensen, 2021; Petersen et al., 2022). However, in the above studies, conversations were initiated using communication tasks (Diapix or puzzle), which required active participation and interactive exchange of information between two interlocutors (Baker and Hazan, 2011; Beechey et al., 2018). In the current study, we investigated whether the conversational dynamics are affected in a similar manner if the conversation is less task-bound and occurs between three interlocutors. Conversations between two NH and one HI interlocutors were conducted at two different noise levels. At the low level of noise, the HI interlocutor was either unaided or aided with an HA, while at the high level of noise, they received omnidirectional or directional sound processing. This unbalanced study design was chosen because previous studies suggested that HA amplification affected the conversational dynamics, specifically the speech levels, when communicating in quiet (Petersen et al., 2022). At high levels of background noise, HA amplification ensures audibility, but not intelligibility, for the HI interlocutor. Hence, the effect of reducing background noise through directional sound processing is investigated at high levels of background noise.

In the current study, increased communication difficulty, caused by hearing impairment, higher noise levels, or suboptimal HA signal processing, is expected to result in 1) longer and 2) more variable FTO values (median and interquartile range), 3) longer turn durations, 4) higher speech levels, and 5) increased speaking time for the HI interlocutor specifically. By asking the interlocutors to subjectively evaluate their active participation in the conversation and their perceived use of listening/talking strategies, it was investigated whether any alterations in the conversational dynamics were perceived or deliberately used by the interlocutors.

Focusing on the conversational dynamics of a group, rather than a two-person conversation, posed some methodological considerations on how to determine the communication states of the conversation and how to account for pauses made within a talker’s own turn. Some of these considerations and post-processing steps applied before extracting the five features of the conversational dynamics listed above are described in the section Quantifying Turn-taking in Group Conversations.

## 2 Methods

### 2.1 Participants

Conversations were recorded from 25 groups of three interlocutors fluent in Danish: One older hearing-impaired (HI) interlocutor, one older normal-hearing (ONH) interlocutor, and one younger normal-hearing (YNH) interlocutor. The HI participants were recruited from an internal database of HI test subjects, while all the NH interlocutors were recruited internally among employees at WS Audiology, Lynge, Denmark. All normal-hearing interlocutors passed a hearing screening at 20 dB HL at 500, 1000, 2000, and 4,000 Hz, except two ONH with a 30-dB HL on one ear at 4000 Hz. All YNH were below 35 years of age (mean = 27.2, sd = 5.2, 14 female participants). All older participants (ONH and HI) were required to be older than 50 years of age, but the HI participants (mean = 75.8, sd = 6.5, 9 female participants) were significantly older than the ONH (mean = 54.8, sd = 3.7, 15 female participants, *t* (48) = −14.1, *p* < 0.001). The YNH participants were significantly younger than the ONH and HI participants (*p* < 0.001).

**Figure 1:**
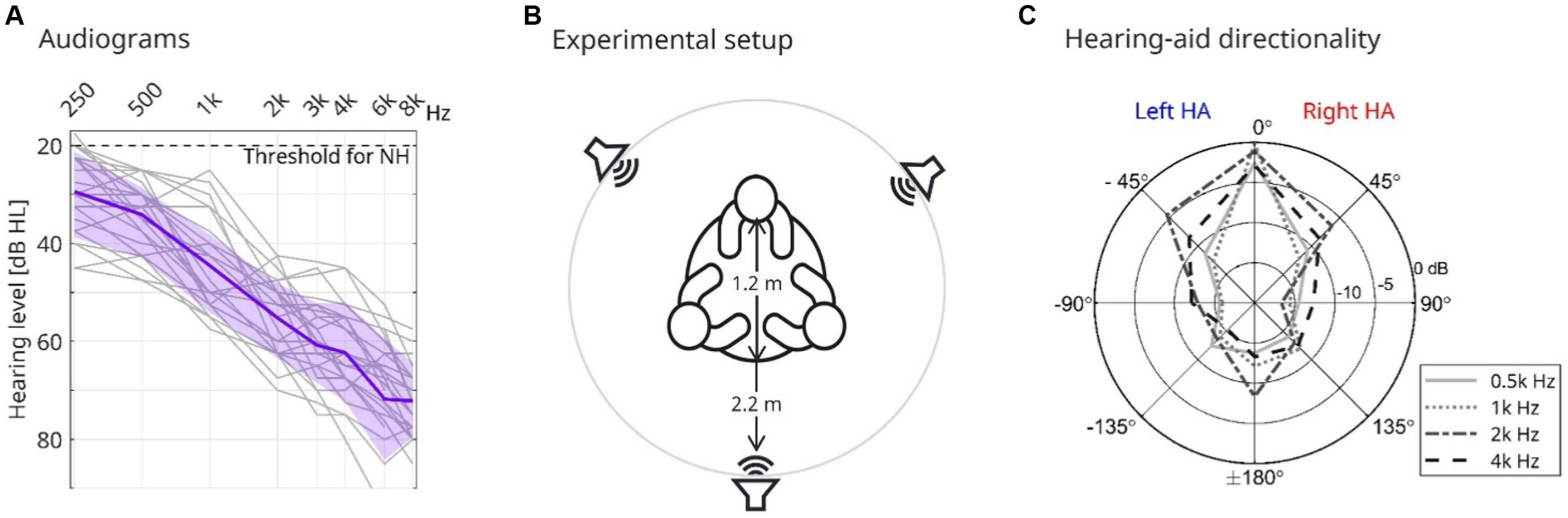
Participants’ audiogram and experimental setup. **(A)** Individual pure-tone hearing thresholds for all HI participants averaged across ears (thin gray lines), participants (bold purple line), and the standard deviation (shaded purple area). **(B)** An experimental setup with the three participants seated equally spaced around a table with a diameter of 1.2 m. A loudspeaker is placed 2.2 m directly in front of each participant. **(C)** Attenuation of white noise when applying the directional sound processing experienced by the HI interlocutor at a high level of background noise (75 dBC, dir condition). The attenuation, in dB, indicated by concentric circles for different frequencies (line types and shading of gray), is shown for different azimuth angles. Note that the attenuations depicted for the negative azimuth angles were recorded from the left HA, while the attenuations at positive angles were recorded from the right HA.

The HI participants had mild-to-moderate symmetrical hearing loss (Figure 1A, pure-tone average across 500, 1,000, 2000, and 4,000 of 48.9 dB HL, sd = 6.1 dB HL) and were experienced hearing-aid users (>1 year of hearing-aid usage).

The triads were grouped at random, ensuring that the YNH and ONH did not work closely together at WS Audiology. Across the 25 triads, 19 had interlocutors of mixed genders, while 2 had only male and 4 only female participants.

All participants gave their written informed consent, and the study was approved by the regional ethics committee (Board of Copenhagen, Denmark, reference H-20068621).

### 2.2 Experimental setup

The experiment was conducted in a meeting room at WS Audiology, with the participants seated at a round table (Figure 1B). The positions of the YNH, ONH, and HI at the table were balanced across triads. Three loudspeakers were placed 2.2 m directly in front of each participant (Figure 1B). The background noise presented by the loudspeakers was spatially recorded noise from the canteen of WS Audiology, which was presented at either 50 or 75 dBC SPL.

The conversations were individually recorded by each interlocutor using a directional headset microphone (DPA 4088, Allerød, Denmark). All sounds were presented and recorded via customized Matlab scripts (2018a) at a sampling frequency of 44.1 kHz. As the headsets were not easily calibrated, individual 5-s speech signals were recorded from the headset, as well as from a calibrated omnidirectional reference microphone (B-5, Behringer, Willich, Germany) placed at the center of the table. Combining the attenuation of the speech signal recorded from the headset to the reference microphone with a calibrated reference signal recorded from the reference microphone, it was possible to compute the conversational speech levels recorded from the headset in dB SPL.

### 2.3 Conversational task

To ensure a natural and free conversation between the three previously unacquainted participants in each triad, two conversational types were used: Consensus questions (e.g., Can you come up with a three-course dinner consisting only of dishes none of you likes) and picture cards with three keywords (e.g., a picture of a crowd at a festival with the keywords festivals, music, and summer). These two ways of initiating a conversation have previously been tested and found to spark natural and balanced conversations between interlocutors (Petersen et al., 2022). The test leader showed the picture or read the consensus question aloud before each 5-min conversation. The pictures and questions could be used to guide the upcoming conversation, but the triads were instructed that deviation from the topic/question was allowed. The participants were not instructed to behave or speak in a particular manner but to act as naturally as possible.

### 2.4 Hearing-aid fitting

The HI participants were equipped with Signia Pure 312 7X receiver-in-the-canal HAs with M-receivers and closed-sleeve instant domes fitted with the NAL-NL2 prescription rule (Keidser et al., 2011). No further fine-tuning, feedback tests, or real-ear measurements were performed. The frequency-based noise reduction system was disabled in the fitting software (Connexx version 9.6.6.488, WS Audiology), and two programs were made: One with omnidirectional and another with directional sound processing.

During conversations with a low level of background noise (50 dBC), the HI interlocutor was either not wearing HAs (denoted the unaided condition) or wearing HAs with omnidirectional sound processing (denoted the aided condition). Before the unaided conversations, the HAs were removed by the test leader in a discrete fashion to avoid notifying the NH conversational partners. Furthermore, the HI participants had been instructed not to notify the NH conversational partners that they were unaided.

During conversations with a high level of background noise (75 dBC), the HAs worn by the HI interlocutors were either providing omnidirectional sound processing (denoted omni, settings identical to the aided condition) or directional sound processing (denoted dir) designed to suppress noise sources based on their spatial position. The directional attenuation pattern was fixed using the Signia App (provided by Sivantos Pte. Ltd), controlled by the test leader, in which the pattern was set to the narrowest beam possible, providing 10–15 dB attenuation of white noise presented from directions beyond +/−45 degrees azimuth (Figure 1C).

### 2.5 Experimental procedure

Before the actual experiment, the triads did two 5-min training conversations. The training served to introduce the two conversational types, to acquaint the participants with each other, and to introduce the background noise used during the experiment. During the first training round, participants were given a consensus question to discuss in quiet; during the second training round, they discussed a picture card in canteen noise presented at 60 dBC, a noise level between the low and high levels of noise used during the actual experiment.

A total of 12 experimental conversations were recorded from each triad in the four different experimental conditions (unaided and aided in low noise and omnidirectional and directional processing in high noise), each repeated three times. The order of the four conditions was balanced within three blocks, while the conversational types were balanced across conditions within each triad. The participants had a mandatory break after six experimental conversations.

After each conversation, all participants provided individual subjective ratings of their active participation and the perceived usage of listening/talking strategies. All participants answered the question, ‘*If the conversation would have taken place in quiet, I would have participated: Put a cross on the scale’*, with the scale ranging from 0 (a lot less active) to 10 (a lot more active), with 5 indicating the same perceived activity level as if the conversation was being held in quiet. The formulation of the second question differed depending on hearing status: Both questions started with ‘*In comparison to a conversation in quiet, to which degree do you feel the noise made you …*’, with the ONH and YNH being asked ‘*change the way you communicated*, e.g.*, by changing the way you expressed yourself, used your voice, or body language?’*, while the formulation to the HI was *‘use listening tactics, such as asking for repeats, asking to speak up or turning your better ear to the speaker?’* For both questions, the scale ranged from 0 (no change) to 10 (a lot of change).

### 2.6 Statistical analysis

The effects of the experimental contrasts on the measures of conversational dynamics were investigated through Linear-Mixed Effects Models (LMERs) using the *lm4* package for *R* (Bates et al., 2015). The experimental design of the current study cannot be treated as a 2×2 design because the HA conditions differ between the noise conditions (low noise levels: unaided and aided/omni; high noise levels: omni/aided and dir). For this reason, it was chosen to test each of the three experimental contrasts (background noise level, providing HA amplification at low noise levels, and providing directional processing at high noise levels) in three separate LMER models.

All models included the fixed effects hearing status (HI, YNH, and ONH), experimental contrasts (two conditions for each contrast, see details below), and their interaction effect, with a random intercept of triad and person varying within the triad, i.e., *x ~ hearing + conditions + hearing:conditions + (1 | triad/person)*. When testing the effect of the experimental contrast background noise (low vs. high levels of noise), the two conditions included were aided and omni. When testing the effect of providing HA amplification during low levels of noise, the conditions were unaided and aided, and finally, the effect of the experimental contrast directional sound processing was investigated by comparing the conditions omni and dir during high levels of background noise. The predicted variable *x* in the statistical model will be the five measures of the conversational dynamics and two subjective ratings of the conversation. The extraction of the five measures of conversational dynamics is described in detail in the following section.

## 3 Quantifying turn-taking in group conversations

The focus of the following section is on the methodological considerations of how to perform voice activity detection, determine the communication states when three interlocutors, instead of two, are interacting, and how to deal with pauses made within one talker’s own turn. The final part of this chapter will provide a detailed description of the features of the conversational dynamics used in the current study.

### 3.1 Voice activity detection of individual interlocutors

Quantifying the conversational dynamics requires knowing when each interlocutor is speaking, e.g., by performing individual voice activity detection (VAD). VAD can be done automatically, either using simple methods based on short-term energy changes and thresholding or using more advanced neural network implementation (Sharma et al., 2022). Accurate VADs are important when computing the features characterizing conversational dynamics to reliably identify the beginning and end of all utterances.

One major issue in the application of automatic VADs is crosstalk, i.e., speech from the conversational partners is audible in the recording of the targeted interlocutor. Due to the distance between talkers and the directionality of the headsets worn by the interlocutors, the amplitude level of the crosstalk is generally lower than speech from the targeted talker. However, natural speech has a large dynamic range. At low noise levels (50 dBC), the speech volume of single utterances ranged from 25.9 to 84.1 dB SPL (across all talkers); however, an average of 12.4% of all intervals without speech (background noise, crosstalk, and artifacts) exceeded the minimum speech level. At the high level of background noise (75 dBC), the speech volume ranged from 33.5 to 88.0 dB SPL, but a significantly lower percentage of the background noise exceeding the minimum speech level (7.8%, *F* (1,877) = 29.3, *p* < 0.001). When testing the performance of various energy-based VAD approaches on data from the current study, this ~10% overlap between targeted interlocutor speech and non-speech caused unreliable VAD detections, including false positives and false negative detections.

For the current study, no automatic algorithm was identified that could provide a reliable VAD detection without erroneously labeling crosstalk as speech or vice versa. Hence, the VAD was performed manually based on the following rules: 1) All utterances should be labeled, including laughing, but excluding breaths and sighs; 2) Pauses between utterances shorter than 180 ms should be marked as speech to avoid cutting off stop closures (Heldner and Edlund, 2010); 3) Utterances shorter than 90 ms should not be marked as it is not assumed to be speech (Heldner and Edlund, 2010).

### 3.2 Determining the conversational states

From the binary output of the individual VADs (1 = interlocutor speech, 0 = not interlocutor speech), the conversational states, i.e., the organization of turns between interlocutors, must be determined before extracting the features of the conversational dynamics.

Before determining the conversational states, all instances of laughter were removed from the output-VAD because laughing does not constitute a wish from the interlocutor to “take the floor” (Heldner and Edlund, 2010). Across all interlocutors, between 0 and 16 instances of laughter were removed per conversation (on average 0.60 laughs/min). Note that since laughing often manifests as short bursts separated by unvoiced silence, consecutive bursts of laughter were grouped into one instance of laughing.

**Figure 2:**
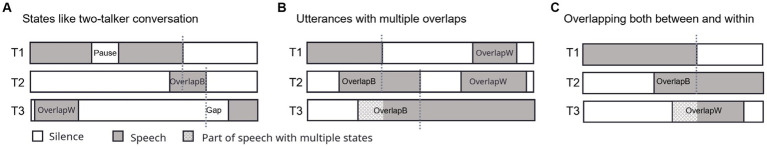
Illustration of conversational states of a three-talker conversation (T1–T3). **(A)** Most states occur between two of the three talkers and are identical to the states observed in two-talker conversations, i.e., the turn-taking happens in an overlap between (overlapB) T1 and T2, in a gap between T2 and T3, or speech (T3) can completely overlap within (overlapW) the turn of another talker (T1). Dotted vertical lines indicate which talker the floor is transferred to and the floor-transfer offset (turn-taking) times of gaps or overlapBs used in the analysis. **(B)** When three talkers consecutively take turns in overlap (overlapBs), it can create instances where one talker (T3) has an overlapB between both remaining talkers (T1, dotted gray area, and T2, gray area). An utterance (T1) can also overlapW speech of remaining talkers (T2 and T3). **(C)** An utterance (T3) can overlapB one talker (T1) but overlapW another (T2) at the same time. In the three examples of **(B,C)**, the final conversational state of an utterance is determined by which talker initiated their utterance first (see details in the text).

Following the procedure proposed by Heldner and Edlund for two-talker conversations (Heldner and Edlund, 2010), it is possible to categorize conversations into the following states (see Figure 2A): A break in a talker’s utterance without a change of turn is called a *pause*, while a turn-taking between talkers (a floor-transfer) can either happen after a *gap* or in an *overlap between* (overlapB) speech. Finally, an utterance can happen simultaneously with an ongoing turn creating an *overlap within* (overlapW) other interlocutors’ turns. This general procedure can also be applied to triadic conversations when only two of the three interlocutors are active. However, if all three interlocutors are active at the same time, the resulting multiple overlaps will cause one utterance to be assigned to multiple conversational states (see text below and Figure 2 for more detail). In the current study, we wish to determine the conversational states of the entire conversation, meaning that each utterance should only have a single conversational state. As detailed below, this requires adding a few exceptions to the procedure proposed by Heldner and Edlund.

Figure 2 illustrates the three examples where overlapping utterances fall into two conversational states. In Figure 2B, Talker 2 and Talker 3 (T2 and T3) start an utterance that overlaps T1 (overlapB). As T3 initiates the utterance later than T2, an additional overlapB between T2 and T3 occurs (indicated with light gray in Figure 2B). In this case, the turn should be transferred from T1 to T2 and then from T2 to T3. The resulting duration of the overlapB included in the analysis of the floor-transfer offsets is indicated with dotted vertical lines in Figure 2. Figure 2B also shows the example of an utterance made by T1 in overlapW with the speech of both T2 and T3. This utterance is classified as one overlapW and is always said to overlap within the speech of the talker who first initiated their turn, in this case, T3 (Figure 2B). It is also possible for an utterance to be classified as both an overlapB and overlapW, as illustrated for T3 in Figure 2C. As T2 initiates a turn first (in an overlapB T1), the utterance by T3 ends up overlapping within (overlapW) the turn of T2 and between (overlapB) the turn of T1. In this case, the utterance of T3 is classified as an overlapW of the speech of T2, as T2 initiated the speech before T3.

Across all the conversations, an average of 1.28 utterances/conversation was corrected for having two overlapBs (illustrated in Figure 2B, range 0–6/conversation). An average of 0.04 utterance/conversation was corrected for multiple overlapWs (range 0–1/conversation, Figure 2B), while an average of 1.24 utterance/conversation was corrected for being overlapB and overlapW (range 0–7/conversation, Figure 2C).

### 3.3 Correcting pauses within turns

**Figure 3:**
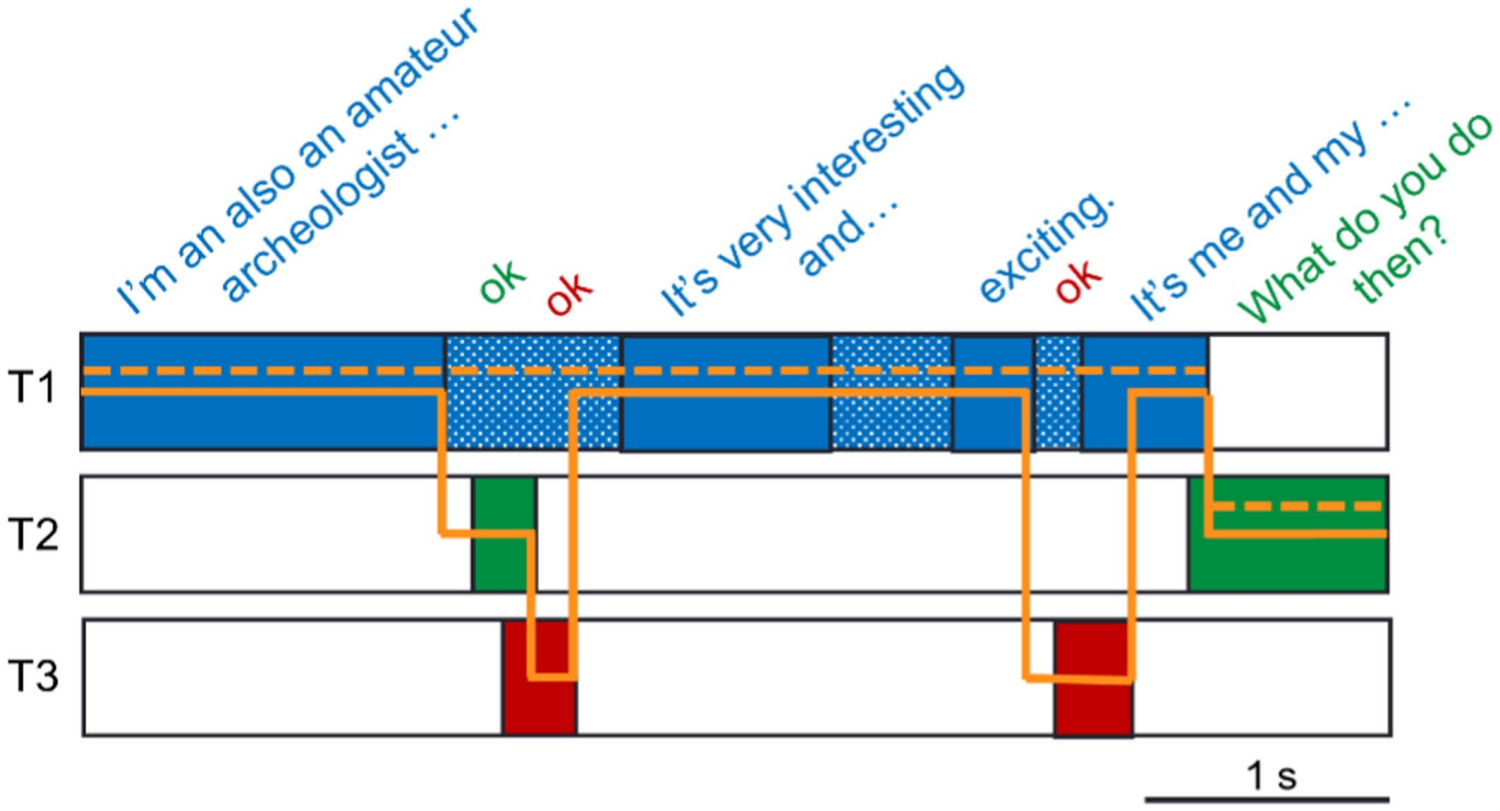
Example of post-processing of turn-taking from a conversation between three talkers (T1–T3). Individual VADs from an excerpt of a conversation (transcription on top) are indicated with fully colored blocks. Based on these, there are six resulting turn-takings (full orange line) between the interlocutors. After post-processing the VADs by bridging pauses within a talker’s own speech shorter than 1 s (dotted blue area), the number of turn-takings is reduced to one, as indicated by the dotted orange line.

Upon inspecting the conversational states and turn-taking resulting from the procedure described in the previous paragraph, it was evident that further processing was needed to capture the dynamics of the conversations. Figure 3 illustrates a typical exchange observed in the triadic conversation: T1 is speaking but receives verbal feedback (denoted backchannels) from both conversational partners (T2 and T3) within natural pauses occurring within the turn of T1. When following the rules for determining the conversational states (see previous section), the example provided in Figure 3 results in six turn-takings (solid orange line). However, considering that the definition of a backchannel is that it does not signal a wish from the talker to take the turn (Yngve, 1970), the timing of backchannels does not have to follow the same social rules as the timing of a turn. Indeed, it has been observed that for utterances made in overlap (overlapW and overlapB), 73% of them are backchannels (Levinson and Torreira, 2015).

To get a better estimate of the true number of turns and their timing in the triadic conversations, post-processing of the output of the VADs was performed to connect utterances constituting a single turn. To this avail, any pauses within a talker’s speech shorter than 1 s were bridged such that the pauses were considered speech. This was done under the assumption that if a talker pauses for less than 1 s, the intention was not to end but to continue the turn. In the example provided in Figure 3, the bridging of pauses reduces the number of turn-takings from six to one.

An average of 7.4 pauses/min were bridged per interlocutor. The conversational states of the post-processed output of the VADs were determined. As expected, bridging the pauses increased the number of utterances overlapping the ongoing turn (overlapW) by an average of 0.60 more overlapW per minute conversation relative to the output of the original VAD.

### 3.4 Features of the conversational dynamics

The dynamics of a conversation can be described by different measures extracted from the individual utterances and conversational states. In the current study, a total of five measures were extracted:

From the individual utterances, the 1) speech levels, defined as the RMS of all utterances, were extracted and scaled using a calibration recording to get the level in dB SPL (Petersen et al., 2022). To avoid including periods of pauses within turns, the speech level was extracted by concatenating utterances of the original VADs, i.e., prior to performing the post-processing described above. From the post-processed individual VADs, the 2) median turn duration was extracted, while 3) the percentage speaking time was extracted as the percentage of the 5-min recording where the interlocutor was talking. As such, the percentage speaking time across the three interlocutors of the conversation can exceed 100% due to overlapB and overlapW.

From the conversational states, the FTOs were extracted by combining gaps and overlapBs to generate the FTO distribution. From the FTO distribution, the 4) median and 5) variability, quantified by the interquartile range (IQR), were extracted as measures of the turn-taking timing.

Furthermore, two subjective evaluations were made for each interlocutor after each conversation regarding 6) the level of activity (participation) and 7) the application of listening (for HI) or talking (YNH and ONH) strategies.

## 4 Results

The fixed effect of hearing status (HI, ONH, and YNH) and experimental contrasts (noise level, HA amplification, and HA directionality) were investigated for the five measures of conversational dynamics and the two subjective ratings made by each interlocutor after each conversation. All statistical results are presented in Table 1. In the visualizations of results, the main effects of conditions and interactions between hearing status and conditions are shown using lines and asterisks, respectively, indicating the level of significance (*** *p* < 0.001, ** *p* < 0.01, * *p* < 0.05).

**Table 1:** Statistically significant effects are highlighted in bold writing. The relevant post-hoc results are presented in italics below the significant fixed effect, indicating the contrasts, estimated difference, and *p*-values.

Experimental contrast (Conditions included)		Noise level (Omni vs Aided)			HA amplification (Unaided vs Aided)			HA directionality (Omni vs Dir)		
	Fixed effect	Statistics		p-value	Statistics		p-value	Statistics		p-value
FTO median	Hearing	F(2,48) = 4.3		**0.02**	F(2,48) = 3.8		**0.03**	F(2,48) = 3.5		**0.04**
		HI – ONH HI – YNH	-85 ms -123 ms	0.06 <0.01	HI – ONH HI – YNH	-79 ms -124 ms	0.08 <0.01	HI – ONH HI – YNH	-79 ms -78ms	0.02 0.02
	Condition	F(1,372) = 2.6		0.11	F(1,372) = 3.4		0.07	F(1,372) = 0.39		0.5
	H:C	F(2,372) = 2.4		0.09	F(2,372) = 2.1		0.1	F(2,372) = 0.29		0.7
FTO IQR	Hearing	F(2,48) = 5.4		**<0.01**	F(2,48) = 5.3		**<0.01**	F(2,48) = 4.7		**0.01**
		HI – ONH HI – YNH	157ms 174 ms	<0.01 <0.01	HI – ONH HI – YNH	107 ms 176 ms	0.054 <0.01	HI – ONH HI – YNH	143 ms 121ms	<0.01 0.02
	Condition	F(1,372) = 0.01		0.90	F(1,372) = 1.0		0.3	F(1,372) = 0.5		0.5
	H:C	F(2,372) = 0.6		0.5	F(2,372) = 0.3		0.7	F(2,372) = 0.2		0.8
Turn duration	Hearing	F(2,48) = 4.7		**0.02**	F(2,48) = 2.8		0.06	F(2,48) = 7.4		**<0.01**
		HI – ONH HI – YNH	719 ms 974 ms	0.03 <0.01				HI – ONH HI – YNH	1325 ms 966 ms	<0.01 0.02
	Condition	F(1,372) = 0.5		0.5	F(1,372) = 4.5		0.03	F(1,372) < 0.001		0.9
					Unaid - Aid	-357 ms	0.04			
	H:C	F(2,372) = 3.3		**0.03**	F(2,372) = 0.9		0.37	F(2,372) = 0.67		0.5
		Omni HI – ONH HI – YNH	1163 ms 1543 ms	<0.01 < 0.001						
Speaking time	Hearing	F(2,48) = 7.7		**<0.001**	F(2,48) = 5.3		**<0.01**	F(2,48) = 8.7		**< 0.01**
		HI – ONH HI – YNH	5% 8.5%	0.02 <0.001	HI – ONH HI – YNH	3.9% 6.7%	0.06 <0.01	HI – ONH HI – YNH ONH - YNH	5.4% 10% 4.6%	0.03 <0.001 0.06
	Condition	F(1,372) = 0.02		0.87	F(1,372) = 0.09		0.8	F(1,372) = 0.01		0.9
	H:C	F(2,372) = 2.4		0.09	F(2,372) = 0.08		0.9	F(2,372) = 0.47		0.6
Speech level	Hearing	F(2,48) =3.7		**0.03**	F(2,48) = 0.8		0.4	F(2,48) = 8.5		**<0.001**
		HI - ONH HI - YNH	-1.8 dB -0.78 dB	<0.01 0.25				HI – ONH HI - YNH	-2.8 dB -2.0 dB	<0.01 <0.01
	Condition	F(1,372) =1366		**<0.001**	F(1,372) = 13.3		**<0.001**	F(1,372) = 8.1		**<0.01**
		Aided - Omni	-8.2 dB	<0.001	Unaid - Aid	0.8 dB	< 0.001	Omni – Dir	0.58 dB	<0.01
	H:C	F(2,372) = 3.1		**0.04**	F(2,372) = 0.8		0.4	F(2,372) = 4.0		**0.02**
		Omni HI – ONH HI - YNH Aided - Omni HI ONH YNH	-2.4 dB -1.3 dB -7.4 dB -8.6 dB -8.5 dB	<0.01 0.07 <0.01 <0.01 <0.01				Omni HI – ONH HI – YNH Dir HI – ONH HI – YNH Omni – Dir HI ONH YNH	-2.4 dB -1.3 dB -3.1 dB -2.8 dB -1.3 dB 0.6 dB -0.1 dB	<0.01 0.07 <0.001 <0.001 <0.001 0.09 0.7
Sub. Rating Participation	Hearing	F(2,72) = 1.5		0.21	F(2,72) = 1.7		0.2	F(2,72) = 0.7		0.5
	Condition	F(1,372) = 19.6		**<0.001**	F(1,372) < 0.01		0.9	F(1,372) = 0.4		0.5
		Aided - Omni	-0.4	<0.001						
	H:C	F(2, 372) = 2.0		0.12	F(2, 372) = 0.5		0.6	F(2,372) = 1.3		0.3
Sub. rating Strategies	Hearing	F(2,48) = 7.1		**<0.01**	F(2,48) = 25.7		**<0.001**	F(2,71) = 0.29		0.7
		HI – ONH HI – YNH	1.5 1.1	<0.01 0.01	HI – ONH HI – YNH	3.1 2.9	<0.001 <0.001			
	Condition	F(1,362) = 536		**<0.001**	F(1,365) = 13.5		**<0.001**	F(1,364) = 0.07		0.8
		Aided - Omni	-4.2	<0.001	Unaid – Aid	0.6	<0.001			
	H:C	F(2,362) = 11.6		**<0.001**	F(2,365) = 7.50		**<0.001**	F(2,364) = 1.6		0.2
		Aided HI – ONH HI – YNH Omni HI – ONH HI – YNH	2.5 2.0 0.5 0.3	<0.001 <0.001 0.2 0.5	Unaid – Aid HI ONH YNH	1.6 0.3 -0.02	<0.001 0.2 0.9			

### 4.1 Floor-transfer offsets

**Figure 4:**
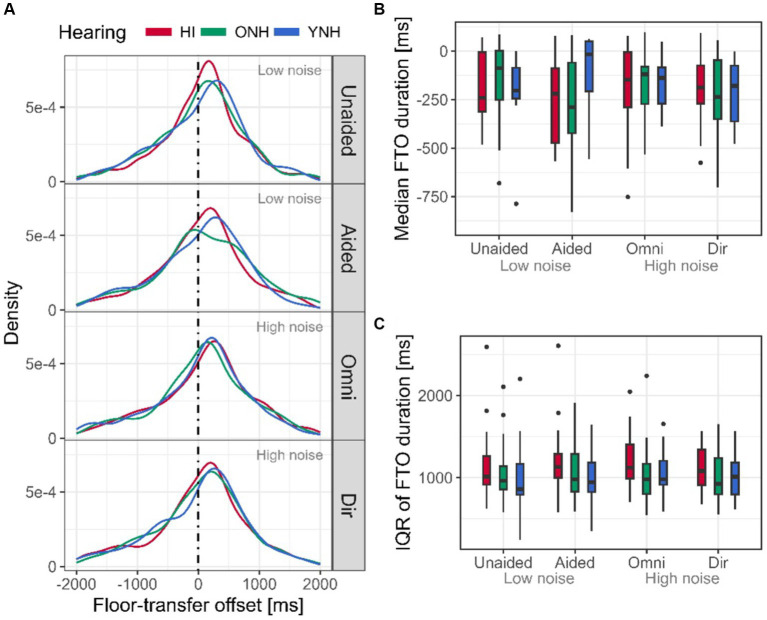
Floor-transfer offset (FTO) distribution and measures. **(A)** FTO distributions for HI (red), ONH (green), and YNH (blue) for all four conditions. Positive FTO values indicate turns initiated after a gap, while a negative value indicates an overlap between turns. The dotted vertical line indicates an FTO of 0 ms, i.e., neither gap nor overlap. **(B)** Median of the FTO distribution extracted for each interlocutor and conditions (averaged across repetitions). **(C)** Variability of the FTO distribution extracted as the interquartile range (IQR) for each interlocutor and conditions (averaged across repetitions). Here and in the following, the boxes indicate the 25th to 75th percentile, and the horizontal lines are the median. Whiskers extend the range of the data, and the dots highlight the outliers.

Across all interlocutors and conditions, the FTO distribution peaked at 208 ms (Figure 4A), i.e., interlocutors tended to start their turn after a short gap. For each interlocutor and conversation, the FTO distribution was formed, and the median and the interquartile range (IQR) were extracted. For all experimental contrasts, a significant effect of hearing status was observed on the median FTO (Table 1; Figure 4B). The HI interlocutors initiated their turns on average 79 ms faster than the YNH and ONH interlocutors at high noise levels. At low levels of noise, the HI interlocutors initiated their turns faster than the YNH (124 ms), but the 79 ms difference between HI and ONH was not significant (*p* = 0.08).

The FTO variability also showed an effect of hearing status (Table 1; Figure 4C), indicating that the spread of the HI interlocutors’ FTO distribution was ~130 ms larger than that of the YNH and ONH interlocutors at both high and low high noise levels, although the difference between HI and ONH in the latter only approached significance (*p* = 0.054).

### 4.2 Turn duration and speaking time

**Figure 5:**
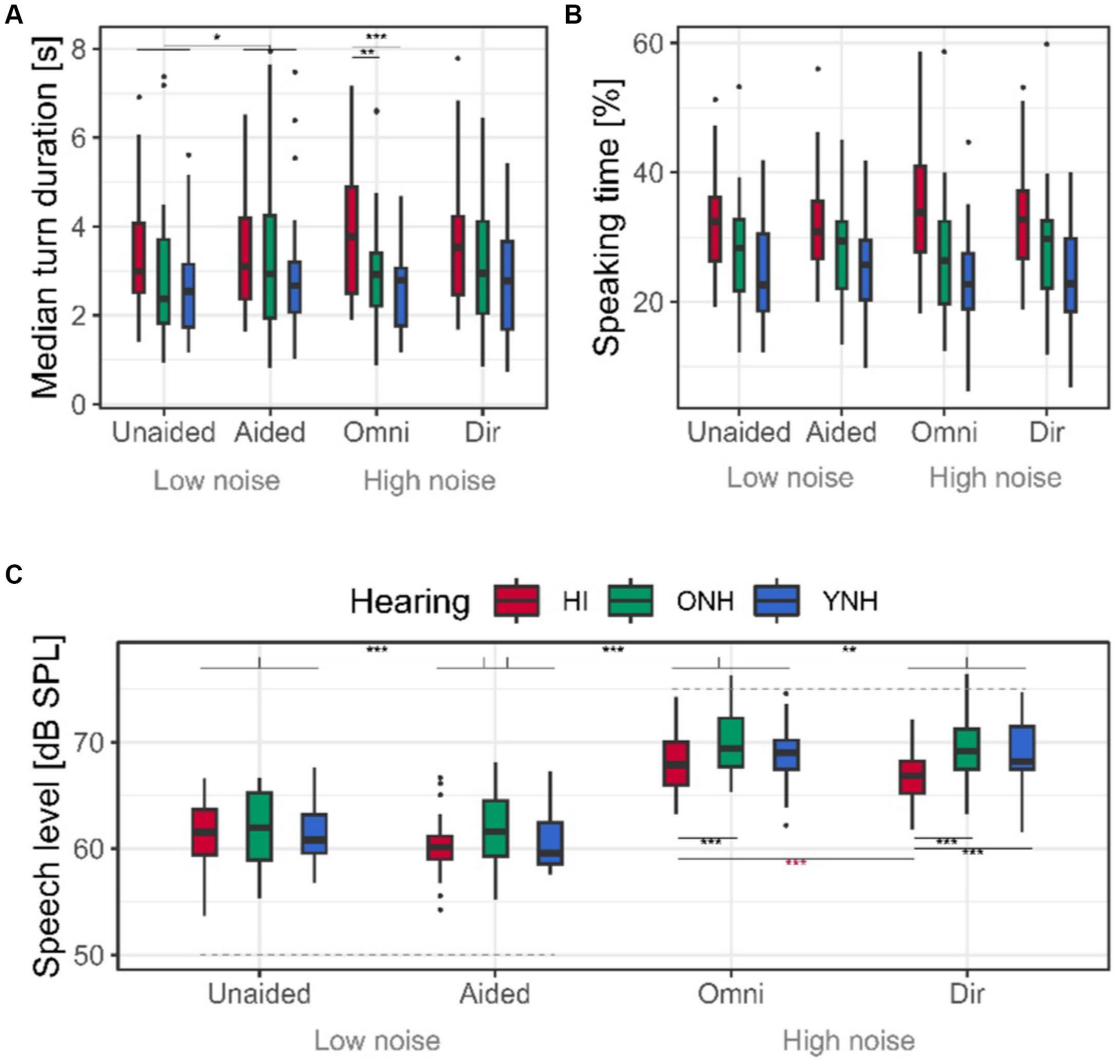
Median turn duration, speaking time, and speech levels. **(A)** Median turn duration resulting from the post-processed VADs across conditions and interlocutor hearing status. **(B)** Percentage of the speaking time of the total conversation duration of 5 min. **(C)** Speech levels in dB SPL. Background noise levels (50 and 75 dBC) of the different conditions are indicated with dotted gray. Asterisks colored according to hearing status indicate the statistically significant results of the *post-hoc* testing of the interaction effect between hearing status and experimental condition.

The median overall turn duration was 3.2 s. The main effect of hearing status in the model testing effect of increasing the noise level (*p* = 0.02, Table 1; Figure 5A) suggests that HI interlocutors spoke in longer turns in general; however, this effect is driven by the significant interaction between noise and hearing (*p* = 0.03), revealing that the HI interlocutors only differ from the YNH and ONH at high levels of noise. This is confirmed by the main effect of hearing status in the conditions with high levels of noise where the HI interlocutors spoke 1.3 s longer than the ONH (*p* < 0.01) and 0.9 s longer than the YNH interlocutors (*p* = 0.02).

No effect of hearing status was found on the turn duration at low background noise, although the result was close to significance (*p* = 0.06). In lower noise, the main effect of HA amplification on turn duration (*p* = 0.03) indicated that all interlocutors’ turns were on average 357 ms longer when the HI interlocutors were aided relative to unaided.

The percentage speaking time was affected by hearing status for all experimental contrasts, indicating that HI interlocutors spoke around 5% more than the ONH and around 8% more than the YNH interlocutors across all conditions (Table 1; Figure 5B). It should be noted that at a low level of background noise, the difference between HI and ONH only approached significance (*p* = 0.06). No difference in the speaking time between the YNH and ONH interlocutors was seen, although there was a non-significant tendency for the ONH to speak more than the YNH in high levels of noise (*p* = 0.06).

### 4.3 Speech level

The conversations held in 50 dBC noise were conducted at an SNR of +11.1 dB on average, while in 75 dBC noise, the SNR was reduced to −6.3 dB when averaging across interlocutors, repetitions, and HA settings.

The speech levels were affected by hearing status at high noise levels but not at low levels of noise (Table 1; Figure 5C). When increasing the noise level (aided vs. omni), the HI interlocutors increased their speech by around 1 dB, which is less than the ONH and YNH interlocutors. Consequently, during the high level of background noise, the ONH spoke 2.4 dB louder than the HI interlocutors (*p* < 0.01), while there was a non-significant trend for the YNH to speak 1.3 dB louder than the HI interlocutor in noise (*p* = 0.07). In terms of SNR, the HI interlocutors talked at −8.0 dB SNR on average at the highest level of noise, while the YNH was speaking at −5.8 dB SNR and the ONH at −5.1 dB SNR.

Significant effects of altering the HA processing were observed. At a low background noise level, all interlocutors spoke 0.8 dB louder when the HI interlocutor was unaided (*p* < 0.001, Table 1). Similarly, all interlocutors spoke on average 0.58 dB louder when the HI interlocutors were listening to the unprocessed omnidirectional sound input (*p* < 0.01). However, a significant interaction effect between hearing status and directional sound processing revealed that while the NH interlocutors generally spoke louder than the HI interlocutors at high noise levels, providing directional sound processing caused the HI interlocutors to reduce their speech level further by 1.3 dB (*p* < 0.001), while the speech levels of the NH interlocutors were unaffected (both *p*’s < 0.09). As a result, the HI interlocutors reduced the SNR experienced by the NH interlocutors from −7.2 dB when listening to omnidirectional sound processing to −8.5 dB when receiving directional sound processing. The HI interlocutors experienced an SNR of −5.5 dB produced by the NH interlocutors in both conditions with high levels of background noise.

### 4.4 Subjective evaluations

After each conversation, the interlocutors were asked to subjectively rate their level of participation as well as their application of listening (for HI interlocutors) and talking (YNH and OHN interlocutors) strategies.

The subjective ratings of the level of participation showed no significant effect on hearing status (Table 1, data not shown, all p’s > 0.2), while increasing the noise level reduced their participation rating by 0.4 points (p < 0.001).

**Figure 6:**
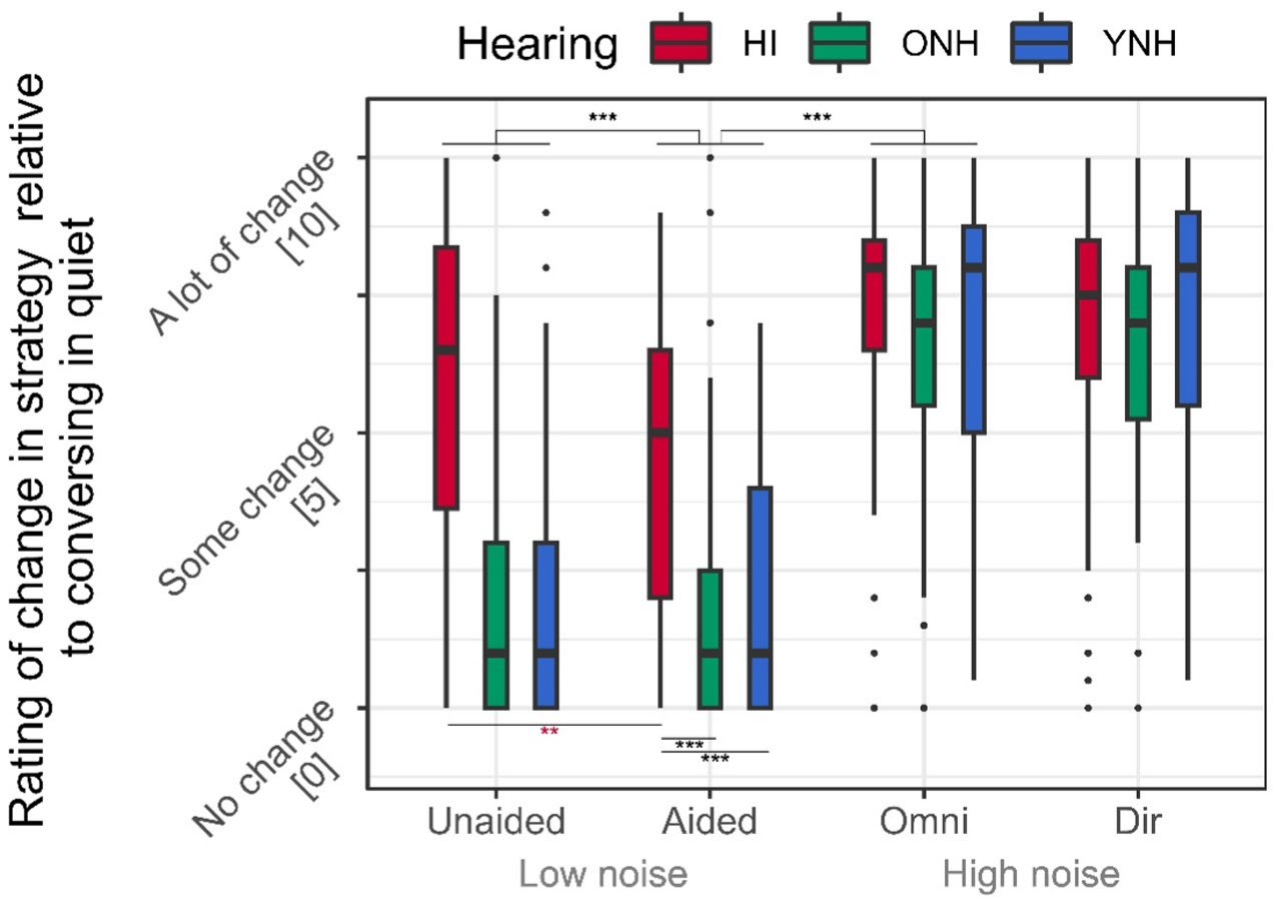
Subjective ratings. The subjective rating of how much the HI rated applying listening strategies relative to whether the conversation had been held in quiet. The NH rated how much they applied communication strategy relative to whether the conversation had been held in quiet. Ratings were performed on a continuous 11-point visual analog scale.

Similarly, the subjective evaluation of the application of listening/talking strategies increased by 4.2 points when the noise level was increased (Table 1; Figures 6, *p* < 0.001). The HI interlocutors rated increasing their usage of listening strategies compared to the application of talking strategies rated by the NH interlocutors when increasing the noise level (Table 1; Figure 6, both *p*’s < 0.05), but the significant interaction effect between hearing status and background noise indicated that hearing status only affected the ratings at the low level of background noise (both *p*’s < 0.001), whereas no differences were observed between HI and NH interlocutors at high noise level (all *p*’s < 0.2).

In low-level noise, the HI interlocutors rated using 3.0 points more strategy on average compared to NH listeners (Table 1; Figures 6, *p* < 0.001). Although a significant main effect of HA amplification (*p* < 0.001) indicated a general 0.6-point decrease in applying strategies when the HI interlocutor was aided, the significant interaction between hearing status and HA amplification (*p* < 0.001) revealed that the effect is driven by the HI interlocutors rating using 1.6 points less strategy when receiving HA amplification (*p* < 0.001), whereas the YNH and ONH rated no changes in their application of talking strategies (both *p*’s > 0.2).

## 5 Discussion

The current study investigated the effect of hearing status and three different experimental contrasts (background noise level, HA amplification, and HA directionality) on the dynamics of a group conversation between one HI and two NH interlocutors. We observed that being hearing impaired affected all measures of conversational dynamics, HA processing, and noise level, which primarily affected speech levels. The following discussion will focus on why the experimental contrasts did not affect the conversational dynamics as hypothesized.

### 5.1 Effect of noise and HA processing on the conversational dynamics

Only a few effects were observed when altering the three experimental contrasts: Increasing the background noise or altering the HI interlocutor’s auditory perception by providing either HA amplification or directional processing.

Beyond increases in speech levels (Figure 5C), the 25 dB increase in the level of the canteen noise did not have any effect on the conversational dynamics (no main effects of aided vs. omni, Table 1). The increased noise level caused interlocutors to speak on average 8.2 dB louder, resulting in a reduction in the communication SNR across interlocutors, from +11.1 dB in 50 dBC background noise to −6.3 dB SNR in 75 dBC background noise. This communication SNR is much in line with a previous study finding that dialogs between an HI and an NH interlocutor happened at −5 dB SNR in 77.3 dBA café noise (Beechey et al., 2020b). For comparison, the standardized Danish speech-in-noise tests find sentence intelligibility (without visual cues) to be lower than 50% for NH listens at −5 dB SNR (Nielsen and Dau, 2009; Bo Nielsen et al., 2014). It should be noted that in realistic everyday listening situations, communication SNRs below +5 dB SNR are rarely observed (Smeds et al., 2015). Nevertheless, the result of the current study suggests that communication at −6.3 dB SNR was possible for both HI and NH interlocutors. This is evident from the fact that the overall percentage speaking time did not alter when increasing the noise level, and the subjective participation ratings only decreased by 0.4 points on the 11-point scale. The neglectable effect of increasing the noise level on the conversational dynamics could be caused by the access to visual cues, the spatial separation of the noise and interlocutors, and/or predictability of the conversational topic. The interlocutors not being able to increase their vocal intensity more, to improve the SNR beyond −6.3 dB, could be caused by the additional physical strain on the vocal cords associated with speaking at higher levels, causing a reduction in voice quality (Södersten et al., 2005). Hence, the SNR of a conversation is likely a balance between speaking loud enough for communication to be successful while at the same time reducing the vocal effort.

As two of the three experimental contrasts (HA amplification and directional processing) were only experienced by the HI interlocutors, it is noteworthy that providing HA amplification affected turn duration and speech level for both the HI and NH interlocutors (Table 1). All interlocutors shortened their turns by 357 ms on average, when the HI interlocutor was unaided (Figure 5A). This observation contradicts the hypothesis that communication difficulty would cause longer turns, as observed with the increased turn duration of the HI interlocutors. The effect of HA amplification on speech level will be discussed in detail in the section Speech Levels are Sensitive to all Experimental Contrasts.

### 5.2 Effects of hearing impairment on conversational dynamics

HI interlocutors were hypothesized to initiate their turns slower and with more variability because their impairment makes them worse at predicting turn-ends than the NH interlocutors (Sørensen et al., 2019; Petersen et al., 2022). Although the HI interlocutors were found to initiate their turns with more variability than the NH interlocutors (higher FTO IQR, Figure 4C), they were also observed to do that faster, not slower, than the NH interlocutors (lower medina FTO, Figure 4B). Previous studies have focused on turn-taking in dyadic conversations; however, the presence of an additional interlocutor adds an element of competition to the conversation. Indeed, the *many minds problem* describes how the complexity and uncertainty of the turn-taking system increase when more than two interlocutors are conversing (Cooney et al., 2020). To ensure getting the turn, interlocutors might be forced to initiate turns earlier in overlapBs. This could explain why the broader FTO distributions in the current study skewed toward negative values (Figure 4A) relative to FTO distributions of the dyadic conversations of previous studies (Figure 2A of Petersen et al., 2022, Figure 4 left in Sørensen et al., 2019). However, it should be noted that although the *many minds problem* can affect turn-taking, the post-processing of the VADs by bridging pauses also has a substantial effect on the turn-taking timing by occasionally causing utterances classified as overlaps within (overlapW) to be bridged with later utterances, resulting in larger negative FTO values (Section 3.2 Correcting Pauses Within Turns). Despite the influence of the post-processing step, it is nevertheless interesting to note that the peak of the overall FTO distribution, at 208 ms, is comparable to that of previous studies (~230 ms in Petersen et al., 2022, ~275 ms in Sørensen et al., 2019), lending more emphasis on the stability of the average turn being taken with a 200-ms gap (Levinson and Torreira, 2015).

When facing difficult communication situations, it has been reported that HI interlocutors can adopt a face-saving strategy of speaking more to avoid listening (Stephens and Zhao, 1996). The HI interlocutors in the current study generally took up around 5% more speaking time relative to the NH interlocutors. Increasing the noise level did not affect the speaking time of the HI interlocutors. This suggests that although the HI interlocutors took up more speaking time, they did not seem to deliberately use the strategy of dominating the conversation to avoid listening when the background noise level increased.

The HI interlocutors also produced longer turns (Figure 5A), although the effect seemed to be largest at higher levels of background noise, as the effect of hearing status was only near-significant at the low noise level (Tables 1, *p* = 0.06). Overall, HI interlocutors spoke for around 1 s longer per turn, which must be considered a substantial increase relative to the overall average turn duration of 3.2 s. The HI interlocutors could have prolonged their turns by edge speaking slower, adding more pauses, or including more filler words such as “*um*” or “*uh*” in their speech. Non-informative filler words play an important role in coordinating turn-taking by helping the interlocutor take the floor fast, or keep the floor, while planning an upcoming utterance (Clark and Fox Tree, 2002). Indeed, it might be speculated that if the longer turn durations observed for the HI interlocutors are caused by uttering filler words, these might cause the faster turn-taking timing (lower FTO median) observed for the HI interlocutor.

It should be noted that the HI interlocutors were significantly older than the two NH groups, which could lead to speculation on whether the observed effect of hearing status was driven by the difference in age between the groups. However, as the ONH interlocutors were also significantly older than the YNH participants, it would be expected that any potential age effects would have resulted in significant differences between the YNH and OHN groups, which was not observed.

### 5.3 Speech levels are sensitive to all experimental contrasts

Similar to a previous study (Petersen et al., 2022), speech level was the measure most affected by alterations in communication difficulty (Table 1 and Figure 5C). At low background noise, hearing status had no differential effect on the speech level; however, when the HI interlocutor did not receive HA amplification (unaided), all interlocutors spoke louder. The observed decrease in speech level of 0.8 dB upon providing HA amplification is comparable to the 1.1 dB decrease in speech level observed when providing amplification to HI interlocutors in dialogs held in quiet (Petersen et al., 2022).

When increasing the level of background noise, all interlocutors increased their speech level. However, the increase was around 2 dB larger for the NH interlocutors than for the HI interlocutors. Again, a similar effect was observed when adding 70 dB background noise to a dialog, in which NH interlocutors increased their speech level by 3.2 dB more than the HI interlocutors (Petersen et al., 2022). Hearing status was found to affect speech level differentially, suggesting that the NH interlocutors made up for the added communication difficulty experienced by the HI interlocutors when communicating in noise by speaking louder. Interestingly, when providing directional sound processing, thereby reducing the noise level experienced by the HI interlocutors, the HI interlocutors reduced their speech level by 1.3 dB, further reducing the SNR experienced by the NH interlocutors. Hence, directional sound processing increased the communication difficulty experienced by the NH interlocutors.

The subjective evaluation of the use of talking strategies during the conversations, including speaking louder, revealed that although speaking louder, the NH interlocutors did not perceive using additional talking strategies when the HI interlocutors were unaided (Table 1; Figure 6). However, the HI interlocutors reported applying more listening strategies when communicating unaided, despite the small increase in speech level made by all interlocutors relative to when the HI interlocutors were aided. At higher levels of background noise, interlocutors reported using more talking/listening strategies. However, it is interesting to note that the additional application of listening strategies in noise rated by the HI interlocutors seemed to match the increase in applied talking strategies made by the NH interlocutors.

The Lombard effect describes the increase in speech level when talking in the presence of noise; however, the effect has rarely been investigated in interactive communication situations. As the findings of the current study highlight, the speech level of interlocutors depends not only on the noise level but also on the communication difficulty experienced by the (HI) conversational partner. Through requests to repeat utterances, statements of not being able to hear, miscommunications, or subtle alterations in facial expressions, gestures, or body posture/movements, such as leaning in or turning the better ear, an interlocutor can influence the conversational partners to increase their speech level. However, the current study also suggests that HI interlocutors alter their speech level according to their own perceived communication difficulty, as evident from the reduced speech level of the HI interlocutors when receiving directional sound processing in a high level of background noise. However, when receiving HA amplification at the lower noise level, the speech levels increased not only for the HI interlocutor but for all interlocutors. During the experiment, the test leader physically removed the HAs as discretely as possible (see section hearing-aid fitting); however, the removed and missing HAs during the unaided condition were visible to the NH interlocutors. It is, therefore, likely that all interlocutors were aware that the HI interlocutor was going to experience communication difficulties in the unaided conditions, potentially causing interlocutors to alter their speech levels going into the conversation. This is contrary to the change in directional sound processing, which was changed through an app, thereby not prompting the interlocutors that the auditory experience of the HI interlocutor was altered.

Altogether, the result of the current study shows that the conversational dynamics of free triadic conversation are relatively stable in response to changes in communication difficulties. This is contrary to previous studies of task-bound dyadic conversations, where researchers found changes in many different measures of conversational dynamics (Hazan et al., 2018; Beechey et al., 2020a,b; Sørensen, 2021; Petersen et al., 2022). We can only speculate what caused the observed stability of the conversational dynamics in the current study: Perhaps the interpersonal coordination of a triadic conversation, caused by the many minds problem, influences the dynamics of the triadic conversation more than, e.g., altering the background noise. Perhaps the free conversations allowed the interlocutors to utilize and modify their word usage, linguistics, or body language to help overcome the increased communication difficulty. It is also possible that the conversational dynamics are determined by the fact that two out of three interlocutors were NH, who are potentially less affected by changes in the noise level. Unfortunately, we cannot know which, if any, of the reasons listed above cause the insensitivity of the features of conversational dynamics to the changes in communication difficulties.

## 6 Conclusion

The current study explored whether the dynamics of a free group conversation were affected by the impaired hearing experienced by one of the three interlocutors and whether noise and hearing-aid signal processing would influence it to the same extent as observed in dyadic conversations. It was hypothesized that any alteration of the communication difficulty (noise level, hearing loss, and HA processing) experienced by one or all interlocutors would affect the five measures of the conversational dynamics (FTO median, FTO IQR, turn duration, speech level, and speaking time). This hypothesis could not be uniformly confirmed: Interlocutors with hearing loss showed the expected larger variability in turn-taking timing (FTO IQR), taking up more speaking time, having longer turn-durations at high noise levels, and resulted in the NH interlocutors speaking louder, especially at low noise levels. However, contrary to the expectations, it was also observed that the HI interlocutors initiated their turns faster (FTO median), not slower, than the NH interlocutors. An overall increase in the noise level of 25 dB SPL caused an increase in the speech levels but did not affect the turn-taking timing, turn duration, or distribution of speaking time. Furthermore, improving listening for the HI interlocutors by providing HA amplification at low noise levels and directional sound processing at high noise levels had no effect on the conversational dynamics beyond the speech level: At low noise levels, providing HA amplification to the HI interlocutors cause all conversation partners to speak at a lower volume. At high noise levels, providing directional sound processing caused the HI interlocutor to speak at a lower volume.

From the current results, the speech levels were observed to be a measure of the conversational dynamics most sensitive to alterations in the communication difficulty experienced by the group (background noise), as well as the HI interlocutor when providing HA amplification and directional sound processing.

## Data availability statement

The original contributions presented in the study are included in the article/supplementary materials, further inquiries can be directed to the corresponding author.

## Ethics statement

The studies involving humans were approved by Board of Copenhagen, Denmark, reference H-20068621. The studies were conducted in accordance with the local legislation and institutional requirements. The participants provided their written informed consent to participate in this study.

## Author contributions

EP: Writing – original draft, Writing – review & editing.
